# Not all crops are equal: the impacts of agricultural investment on job creation by crop type and investor type

**DOI:** 10.1016/j.heliyon.2022.e09851

**Published:** 2022-07-04

**Authors:** Habiba Mohammed Yimam, Logan Cochrane, Melisew Dejene Lemma

**Affiliations:** a Institute of Policy and Development Research, Hawassa University, Ethiopia; b HBKU (College of Public Policy), Qatar; c Carleton University (Global and International Studies), Canada; d Hawassa University (Institute of Policy and Development Research), Ethiopia; e Institute of Policy and Development Research (IPDR) and Department of Journalism, Hawassa University, Ethiopia

**Keywords:** Foreign direct investment, Job creation, Agricultural investment, Africa, Ethiopia

## Abstract

Significant agricultural investment has taken place across Africa over the most recent two decades. An expanding set of literature analyzes these investments, often using case study and comparative approaches. While this is important, not all agricultural investments are equal, yet they are often described as being such. Few studies utilize large data sets to conduct quantitative, comparative research. This paper examines investments in Ethiopia, using quantitative data of 102 investments that took place in one region between 1998 and 2018. Using this unique dataset, we conduct a comparative assessment of investments, analyzing traits such as crop choice, job creation, job type, implementation status, and investor type (Ethiopian, foreign, diaspora). We find that Ethiopian investors are granted larger average leases of land, though are fewer in number in comparison to foreign and diaspora investors. The same category of investors (Ethiopians) have higher implementation status but have created the least per hectare permanent and seasonal jobs. The regression analysis, however, shows that there is no statistically significant difference among the three types of investors in terms of per hectare job creation. From the investment types, horticulture/flowers created the most employment per hectare, followed by vegetables and fruits production. This evidence contests common narratives about agricultural investment and provides a basis for decision makers to better enable positive outcomes, such as greater job creation.

## 1 Introduction

Since 2008, African countries have transferred large tracts of land to investors of both national and international origin. As reported by the World Bank (2010) the amount of land transferred to foreign investors in 2009 alone was estimated to be up to 50 million ha, whereas on average only 4 million ha had been transferred annually before 2008. This was reflective of a global trend. A couple of years following the 2009 rush for land, Oxfam (2011) reported that throughout the Global South a total 227 million hectares had been granted to foreign investors. Ethiopia, being among the top African recipients of foreign direct investment (FDI) in agriculture, leased between 0.6 million and 3.6 million ha during these years (Lorenzo et al., 2009; Abbink, 2011; Rahmato, 2011; The Oakland Institute, 2011; Yassin, 2014). There is significant variation in reporting of the scale of investments, largely due to a lack of data transparency. The problem of data accuracy (and particularly its incompleteness) in Ethiopia has been detailed by Cochrane and Legault (2020). This paper draws on a unique dataset obtained from a regional investment commission in Ethiopia, addressing many of those data uncertainties. Using this dataset, we conduct a comparative assessment of investments, highlighting the unique traits of investment and their impacts, including crop choice, job creation, job type, implementation status, and investor type (Ethiopian, diaspora, foreign).

The drivers of the rush for land across Africa have varied over time: in some countries, foreign land holdings were acquired during the colonial era, some lands were taken by governments to create state-run farms and later privatized, in yet other countries liberalization brought about by structural adjustment in the 1980s enabled foreign investment (see collection of national case studies in Cochrane and Andrews, 2021). However, the ‘triple crisis’ of food, fuel and finance in 2007/08 was the most recent driver of a large rush for agricultural land, in the tens of millions of hectares. As with Dejene and Cochrane (2021), we do not adopt the term ‘land grab’ or ‘large scale land acquisition’, as these imply illegality and legality respectively. Instead, we refer to the ‘land rush’ and prefer that each investment be analyzed individually, and assessments made accordingly (such assessments are not simply ones of contractual legality per se, but may also include aspects of duress in land expropriations affecting free and informed consent.

The Ethiopian experience of the global land rush began before the triple crises of 2007/08, in a crop-specific rush for land: investment in the fresh cut flower industry, with fresh cut flowers being added as a key export commodity in the 2003/04 annual report from the National Bank of Ethiopia. This was followed by investments in food commodities and biofuels, both driven by external factors, the former in seeking to expand corporate farm-to-plate supply chains and the latter to meet biofuel targets, particularly for the European Union (Yassin, 2014; Cochrane and Adam, 2017).

Ethiopia, being at the forefront of African countries promoting agricultural investment, and being among the top recipients of FDI in the agricultural sector (Dheressa, 2013), is an important country for analyzing the quantitative data and long-term trends. Ethiopia has attracted a diversity of investors and investment types, thereby offering an opportunity to conduct comparative assessments regarding the unique traits of investments to explore their diverse impacts. One of the factors of focus in this analysis is job creation, as this has been listed by governments as a justification to expropriate land and has been touted by investors and development banks as key to the ‘win-win-win’ framing of agricultural investment. According to Alamirew et al. (2015), creating employment opportunities is among the most commonly mentioned expected benefits of the Ethiopian government from agricultural investments. Among the primary objectives cited by the Ministry of Agriculture and Rural Development (MoARD) is creating employment opportunities, alongside expanding agricultural exports and the development of infrastructure (Rahmato, 2011). Although there are investment-specific case studies (e.g. Aabø and Kring, 2012) and papers that discuss job creation (e.g. Alamirew et al., 2015; Philipp et al., 2015; Ali et al., 2017), few assessments have been able to conduct comparative studies using a large data set of investments to assess the variation of impacts based on factors such as crop type and investor type. This paper draws on a unique government data set from one region of Ethiopia to contribute such an analysis.

## 2 Materials and methods

Our analysis draws on a unique data set that we have obtained from the former Southern Nations, Nationalities and Peoples (SNNP) Regional State of Ethiopia (before the separation of Sidama Regional State and the South West Regional State). This region is one of the most heavily impacted by the land rush in terms of total hectares leased as well as hectares leased adjusted to the size of the region (Lavers, 2016; Dejene and Cochrane, 2021). The data set was obtained and updated in 2020, during the same period that the new Sidama Regional State was formed, which was then a part of SNNP. Our data is reflective of SNNP previous to the formation of Sidama Regional State, inclusive of what is now Sidama Regional State and South West Regional State. Using data obtained from the regional investment office, which was updated and expanded, we developed a database that comprises 102 agricultural investments. The expansion of the data set added investment attributes such as the country of origin of investors, location of the investments, the year when the contracts were signed, the degree of implementation, the size of land granted and developed, the types of crops harvested, and the number of seasonal and permanent jobs created.

To conduct the analysis, we utilized a database of agricultural investments of the regional state where the study is conducted. That makes the current study different from others that either rely on secondary data, such as data from the Land Matrix, or use data collected from a few case study investment sites. The data that is found on the Land Matrix, the most widely used database by researchers, as reported by Cochrane and Legault (2020), can only be taken as indicative of what is going on rather than as a complete and reliable data source. Comparisons between the Land Matrix data with government data, find the former incomplete, not up-to-date, and missing many of the smaller and domestic/diaspora investments (Cochrane and Legault, 2020). These problems are overcome with the database we developed, as our data is up to date (with site verifications), includes both small and large investments and all types of investors that are designated by the government as Ethiopian, diaspora, and foreign investors.

We use the database to examine the nature of agricultural investments in the region and to conduct a regression analysis on the relationship between the number of jobs created (permanent jobs, as the duration of seasonal jobs is not included), investor type, and crop types. The assumption behind the regression analysis is that not all agricultural investments are equal, yet there is a paucity of data to disaggregate and assess the variables to identify trends. One motivation to conduct this study, although not the only one, is an expressed interest from the Ethiopian investment commission to have evidence to support better-informed decision making. The government request for evidence emerges out of government frustration, whereby many investors are not utilizing the leased lands, resulting in some land deals being cancelled, others having their land reduced in size, and in yet others fines or threats of fines have been levied for companies that are not abiding by the contractual agreements. Relatively more is known about failed investments, as these have drawn more journalistic and research attention, however relatively little is known what enables more positive outcomes (as viewed by government and/or community members), particularly with regard to employment creation. To provide some evidence regarding this, we try to identify which investors and which crop types might be considered by some as having more positive outcomes (e.g. creating a larger number of permanent jobs).

For the analysis we adopted Negative Binomial Regression, a kind of Poisson Regression, for the reason that the dependent variable (i.e. the number of jobs created) is count data. We sorted investors into three groups as Ethiopian, diaspora, and foreign (adopting the government’s classification of such) and we organized the crop types into nine categories. We then checked the requirements for the model before running the regression analysis, being that the dependent variable should be a count data and there has to be one or more independent variables, both were met and we proceeded to use the model.

We utilized SPSS version 26, to conduct negative Binomial regression. What follows is a theoretical background of the model employed and the reasons behind employing this particular model. A Poisson regression is used for analysis of nonlinear distributions, particularly wherein the data is appropriate for discrete/count data, under the condition that the mean of the dependent variable should be equal to its variance. In reality, an assumption that the mean equals the variance (known as equi-dispersion) infrequently works. In most cases the variance of count data is greater than the mean (over-dispersion). In that case, the use of a negative binomial model is more appropriate, since it is made to handle the problem of over-dispersion (Georges and Charles, 2014). Negative binomial regression is inclusive of a random component, which allows for the analysis to consider the uncertainty regarding the actual rates for which events occur. In other words, it is an extension of the Poisson regression model that adds an ancillary parameter that allows over dispersion, as shown in the following Eq. (1). For these reasons, it is the standard method that is used to model over-dispersed Poisson data. As a result for the negative binomial probability distribution is:

(1) δ^2^ = μ + μ^2^/r; μ > 0, r > 0

where:

δ2 is variance,
μ is the mean and
r is ancillary parameter of the model.

And the Negative binomial regression model is written as:

(2) Ln μ_i_ hat = β_0_ + Σ^p^_j=1_β_i_ X_ij_

where: i= 1,2,… n, j= 1,2,…p, p is the number of independent variables, β is unknown parameter, and a set of x (independent variables) (William et al., 1995). The above Eq. (2) shows the use of the natural log of the dependent variable in the case of over-dispersed data.

The dependent variable of the regression analysis is the number of permanent jobs created, whereas we use two categorical independent variables. The first being the types of investors-grouped into Ethiopian, diaspora and foreign. The second independent variable is the types of crops harvested by the investors. Nine different crop categories are included in the analysis, based on the naming and categorization of the regional investment bureau, from where we got the data. The size of developed land is used in the analysis as a covariate variable, as it gives us the average number of jobs created by each farm.

Having the number of permanent jobs creation as dependent variable and investor and crop types as predictors, the analysis is conducted with the following hypothesis. The alternative hypothesis/research hypothesis (H_a_) is there is difference among investors and crop types in terms of permanent job creation. Whereas, the null hypothesis (H_0_) is that there is no difference among the three groups of investors and the nine types of crops in terms of the number of permanent job creation. And the output of the negative binomial regression, its interpretation with discussion is presented at the end of the paper-next to the descriptive analysis of the data.

In addition to having a unique dataset, another significant addition of this study is that it is the first paper, to our knowledge, to conduct inferential analysis of factors determining the number of jobs created by agricultural investments in Ethiopia.

This paper addresses two limitations in the existing literature, as identified by Cochrane and Legault (2020). First, they claim that one of the main reasons why the findings of studies on impacts of agricultural investments are mostly negative is because of a potential site selection bias, as the literature is predominated by case studies and wherein the selected sites tend to be either failed or contested. In the current study we include all the agricultural investments of the region and thereby this selection bias is reduced (but not eliminated, as some older, failed and closed investments will not appear in the data provided by the investment commission). Second, one of the missing issues in research on agricultural investments in Ethiopia is diaspora investors. This is somewhat unique to the Ethiopian context, as Ethiopia does not allow dual nationality, but offers a distinct status for people of Ethiopian origin (and incentives for these individuals to invest). In past research, when domestic investors were considered, rarely have these been separated by Ethiopian nationals as domestic investors and foreign nationals of Ethiopian origin who are investing with a diaspora status. This research gap is addressed with in this study. That said, there are limitations to this study that are worth keeping in mind. One limitation is that our data set only covers one regional state, while the findings are informative regarding trends; they also have limited generalizability to other agro-ecological and political contexts. The second limitation is that while the quantitative data was verified, we do not have supporting qualitative data to enrich the contextual information about all of the 102 investments.

The reliance upon a quantitative approach presents some limitations, as the specific context of investors, investments and crops are not included. An example of this is that one crop might be successful in one area, and appears in the data set as such, while in another it fails; this may not be the crop or the investor per se, it may also be the soil type or elevation. Future research is needed to address these particularities to better analyze causes for failure and enablers for more successful agricultural investments.

## 3 Agricultural investments in Ethiopia

Since the early 1900s, governments of Ethiopia have attempted to ‘modernize’ the agricultural sector, as a means to strengthen food security and enable economic transformation (Belay, 2003; Ali et al., 2017). The recent history of large-scale commercial farming in Ethiopia, however, which started in the mid-1990s, can be divided into three phases, as outlined by Rahmato (2014). The first phase (between the mid-1990s and 2000) was characterized by smallscale farms and domestic investors. The second phase (from 2001 to 2007), during which time the investment proclamation of 2003 was issued, was a period that witnessed a boom in the horticultural sector and an increase in the number of foreign and diaspora investors, with the size of land transferred ranging from small to medium scale. The third phase (from 2008 to 2011) was a period of a massive rush for land by investors, while the very large deals have attracted relatively more journalist attention, less attention has been paid to the many (hundreds) of smaller land deals. Following this, a fourth phase can be added, which is the post-2012 period, wherein new policies restricted foreign investment and mega deals, in their place a larger number of small and medium deals occurred, with domestic and diaspora investors returning to the fore (for details on the policy shifts, see Dejene and Cochrane, 2021).

When the new EPRDF government took power in the early 1990s, it appeared to be pro-peasant, as least at the beginning. Exemplary of this was the rapid expansion of agricultural support services for smallholder farmers and in parallel a rapid expansion of agricultural extension workers. However, at the same time, there was also a stated interest in commercialization and large-scale agriculture, which was reflected in one of the earliest policies, the Agricultural Development-Led Industrialization of (1996) (Abbink, 2011). In analyzing the recent history of commercial agriculture in Ethiopia, many writers suggest that the shift of attention of the Ethiopian government from smallholder farming to commercial agriculture became clear in the beginning of 2000s (e.g. Abbink, 2011; Lorenzo et al., 2011; Emma and Vincent, 2018; Dejene and Cochrane, 2021). The investment proclamation, which was prepared in 2003, was the first policy document that codified this policy change, wherein the government assumed (or at least advocated that) foreign investment in the agricultural sector would provide capital and capacity for the governmental modernization objective. Embedded within the assumptions of this transformation was that small-scale subsistence farming would transition to larger, commercial operations. In the same policy document, which would be recognized as incorrect only later on, the Ethiopian government held an assumption that foreign investors would had the potential to not only bring capital and capacity, but also introduce new technologies and connect the agricultural system to global supply chains, and therefore should be promoted and incentivized to invest in the Ethiopian agricultural sector (Rahmato, 2011).

In 2003, another policy document was produced by the government, the Rural Development Policy and Strategy as well as the Plan for Accelerated and Sustainable Development to End Poverty (2005/06–2009/10). Together, these documents can be considered as a critical turning point for the promotion of agricultural investment since they marked the first formalizations of large-scale land acquisitions. The growth and Transformation Plan (GTP I), which began its implementation in 2005, was the next policy document to publicize the interest of the government to shift its emphasis to commercial farming and specifically outlines the development of biofuels as a priority of the country (Abbink, 2011). Notably, this occurred many years before the European Union biofuel targets were put in place, which occurred in 2009, with the Renewable Energy Directive. All these policy changes created an enabling legal and regulatory context for investors to identify Ethiopia as a place to invest when the 2007/08 triple crisis occurred.

It was not just an enabling legal and regulatory environment that attracted investors, however. The Government of Ethiopia, including the Prime Minister, were actively promoting agricultural investment and offering packages of incentives to do so. Amidst these promotional campaigns, it was claimed that Ethiopia had ample potential for agricultural investment with a large amounts of unused or underutilized land. Potential investors were offered a range of incentives to attract the to the Ethiopian agricultural sector (e.g., import tax and custom payment exemptions, the ability to export directly without value addition processes, amongst others; The Oakland Institute, 2011). The government also argued that the expansion of commercial farming in Ethiopia could be achieved without endangering the livelihoods of the local population. This highlights the role played by governments of African countries, as the rush for land of transnational companies was in some cases sought after and welcomed by these governments. Leaders of the countries seeking such investment see the land rush, and attention directed at the agricultural sector, with an optimistic lens (capital, capacity, technology, job creation, poverty alleviation, amongst others; Lorenzo et al., 2011; Lavers, 2012; George and Maru, 2014). As noted, in Ethiopia the practice of leasing land to investors began well before the global 2007/08 commodity price spike, which is usually associated with growing investor interest in farmland around the world (Dejene and Cochrane, 2021). The combination of all these factors enabled the transfer of millions of hectares of land for agricultural investments following the triple crisis (Lavers, 2016).

Due to the federal structure of Ethiopian governance, and the implicit competition between regional states to attract investors to their respective regions (with some undesirable outcomes, such as investors being drawn to the offer of lower prices and better incentives but not to focus on suitability for the proposed investments), the federal government intervened in 2008 and forced the regional governments to hand over their negotiation rights to the federal government for any land lease greater than 1000 ha. This centralization of power was justified as an attempt to reduce corruption related to revenues from leasing land (Moreda, 2018). In 2009, the Agricultural Investment Support Directorate (AISD) was created within the Ministry of Agriculture and Rural Development (MoARD) with the mandate to assist investors and facilitate the land transfer process. The AISD established a Land Bank, which held lists of ‘available’ agricultural land for investment. Regional governments were instructed to identify suitable land and earmark them for agricultural investment opportunities for this purpose (Wubneh, 2018). Commodity and crop specific promotions were also developed, including one in 2012 for palm oil production, produced by the Ethiopian Investment Agency (EIA) (Rahmato, 2014). This 2012 document, however, reoriented the focus to domestic investors, which was a signal for broader changes that would occur in that same year.

After the rush of investments in the agricultural sector between 2007 and 2011, the MoARD (amongst other entities, including banks providing financing for these projects) began to question the viability of many agricultural investments. It also experienced contestation and opposition to the deals being made. As a result, in 2012, the MoARD halted the leasing of land, until a review of the program was conducted. The review found that changes were required, in the processes as well as the institutional arrangements enabling these leases. As a result, a new entity, the Agricultural Investment Land Administration Agency, was established in 2013, which had the mandate to enhance the governance of land leases, including the management of all investment land and administration of Agricultural Economy zones (where the government was to provide basic infrastructures in the zones and lease the land for a much higher rent) as well as scaling down of the size of land transferred (Dejene and Cochrane, 2021).

Also in 2012, an Investment Proclamation introduced new restrictions on foreign investors, putting a ban on the export of raw agricultural products, limiting the maximum size of land leased to foreign investors and granting priority to domestic investors. In the years that followed the AISD and regional governments withdrew and/or cancelled some of the leases granted to foreign investors, primarily due to the fact that investors were not using the land in accordance with the terms for which is was acquired and/or contractual expectations not being met (Dejene and Cochrane, 2021).

## 4 Agricultural investments in SNNP

SNNP is one of the regional states in Ethiopia, among the largest in size and, home to the greatest socio-cultural and linguistic diversity in the country. When Lavers (2012) published his research on agricultural investments, SNNP had experienced the third highest level of investment, in comparison to other regional states. According to the Land Matrix data analyzed by Dejene and Cochrane (2021), SNNP had become the regional state with the most amount of contracted leased land for agricultural investment, and further, as a percentage of land leased SNNP also ranked the highest of all regions. This suggests that SNNP has become a focal target for investment in the last decade and makes it particularly important for analysis. Before we move into the analysis, the following outlines the nature of agricultural investment in SNNP, according to our database of 102 investments (data obtained from the government and then validated and expanded).

As of 2020, when the database was prepared, the total size of land transferred to investors in the region was 75,259 ha. The largest size of land given to a single investor was 10,000 ha and the smallest was 1 ha, with the average size of land granted to investors being 737.84 ha. As discussed by Cochrane and Legault (2020), because this data is sourced from a regional government, contracts handled by the federal government appear to be missed, which explains the discrepancy of data that Cochrane and Legault (2020) find, who rely on the Land Matrix as a data source. The implication here is that a much larger amount of leased land has been leased (had the federal investment commission data also been obtained). A second explanation for the discrepancy is that the Land Matrix has fewer updates regarding reduced or annulled lease contracts, while the government data was up to date in that regard (which we also verified). For researchers, there are two key lessons from this: (1) that the Land Matrix underrepresents some deals, particularly the smaller and domestic/diaspora investments, but also that (2) the Land Matrix over represents some deals due to lack of up-to-date information on reduced and annulled leases. For example, the Land Matrix lists a 40,000 ha Italian investment in South Omo, which was abandoned in 2015, but remains in the database as a contracted lease, contributing to an overrepresented figure, whereas the Land Matrix is missing the majority of land deals contracted in SNNP, according to government data (some of which are excluded by Land Matrix criteria, which only include investments above 200 ha; Cochrane and Legault, 2020). The data held by the investment commission in SNNP, for the active leases it oversees, the total leased land is 75,259 ha (see Table 1), which is what is utilized for this study (see Table 2).

**Table 1:** Land size by investor type.

Investor type	Freq.	%	Average acquired land (in hectare)	Total acquired land (in hectare)
Ethiopian	32	31.1	1,022.1	32,707
Diaspora	13	12.6	690.8	8981
Foreign	57	55.3	588.97	33,571
Total	102	100	737.84	75,259

**Table 2:** Descriptive statistical data of permanent job creation.

Statistics		
The Number of Permanent Jobs Created		
No	Valid	120
	Missing	
Mean		59.8
Median		9.00
Mode		0
Std. Deviation		218.318
Variance		47662.831
Minimum		0
Maximum		2001
Sum		6118

The data shows that in SNNP Ethiopian investors are fewer in number but have acquired larger average land leases, which is contrary to what is commonly believed about the distribution of investors and the size of land they acquired. A good example of this contradiction is the claim made by Dejene and Cochrane (2021), where they suggest that the majority of investors in Ethiopia are domestic, but that foreign investors have acquired much larger contracts. This is not necessarily incorrect, as older or larger leases contracted by the federal government may not be accurately portrayed in the regional state data set, and may be the case in other regions of Ethiopia. Nonetheless, the available data from the regional state government is similar to that of Alamirew et al. (2015), who identified that in some parts of Ethiopia, domestic or diaspora investors are dominant in terms of leased land and the number of investors.

We noted the importance of disaggregating Ethiopian nationals and diaspora investments (foreigners of Ethiopian origin with a unique investor status). A comparison of the three types of investors shows that Ethiopian investors have the highest average size of granted land i.e. 1,022.1 ha followed by diaspora investors at 690.8 ha. In terms of the overall number of investments, the majority were contracted by foreigners, which results in this investor type holding the largest total size of land (as a collective group). Notable here are the diaspora investors as they are largely missing in the literature, or merged into another investor type category. As a result, diaspora investors have not been given enough attention by researchers, as claimed by Cochrane and Legault (2020). The substantial contribution of diaspora investors was also reported more than a decade ago by The Oakland Institute (2011), with recognition of the minimal attention received from academia. However, this remains under-studied.

**Figure 1:**
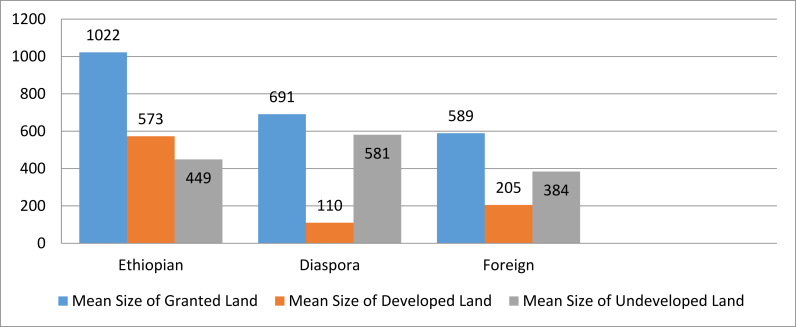
Average size of land granted, developed and undeveloped.

Not all land granted is developed, however. Figure 1 shows the mean size of land granted, developed land and undeveloped land for the three groups of investors. The Ethiopian investors, though smaller in number, were granted the largest average size of land and developed the largest average size of that acquired land. As mentioned above, the mean size of land given to Ethiopian investors is 1,022.1 ha, while it is only 589 for foreign investors, even smaller than the diaspora investor group average. The average size/percentage of land developed by Ethiopian investors was the highest (573 ha, or 56%) amongst the three investors categories, followed by foreign investors (205 ha, or 35%), and then diaspora investors (110 ha, or 16%). While these are only averages, the implication is counter to the broader narrative: domestic investors are getting larger land leases and are developing more land; while foreign investors are granted smaller leases than diaspora investors, they develop more of that land.

This finding differs from what is commonly reported by other researchers, where they claim that larger leases of land are given to foreign investors (Alamirew et al., 2015; Dereje et al., 2017). Although more research is needed, a second implication is that Ethiopian investors are more successful, on average, at developing the land that is acquired. This might be due to their higher levels of understanding of the socio-cultural and political context within which they operate, and/or may have implications about cohesion within community. These are claims that require evidence to assess, which we hope future research can shed light upon.

**Figure 2:**
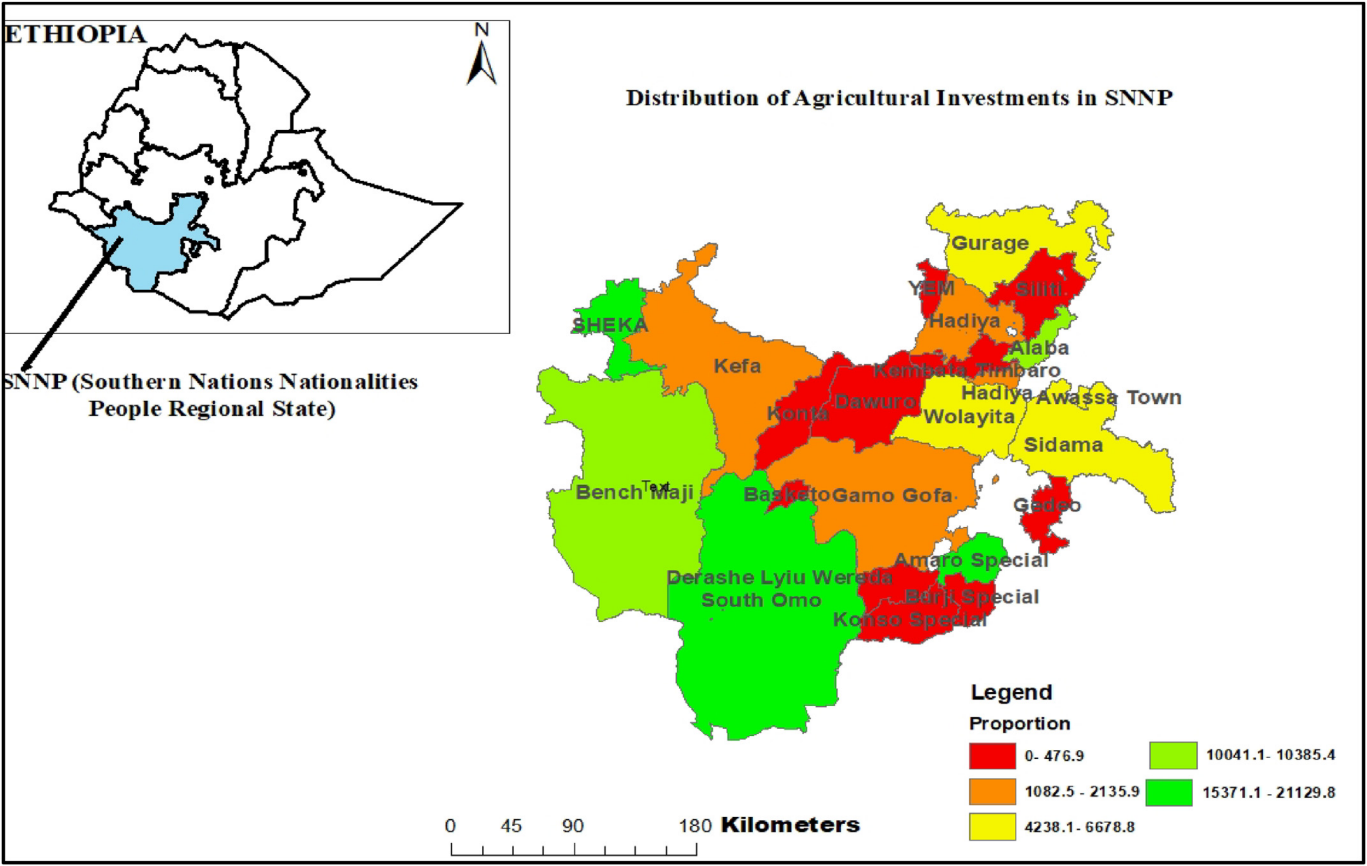
Study Area Map and Distribution of Agricultural Investments in the Region (Scaled by Investments per ha of Administrative Area).

Another contextual factor regarding agricultural investments in SNNP is where the investments take place. As shown in Figure 2, not all administrative zones and woredas (districts) are equally engaging with agricultural investments. In order to show the geographic distribution of agricultural investments in the region, we computed first the ratio between the total land leased to investors and the total size of each zone and special woreda (district) in the region. Then, since the resulting figures are very small we multiplied them by 1,000,000. From this assessment, zones like South Omo, Sheka, and Amaro Special woreda (district) are found to have the highest concentration of agricultural investments, which are areas that are lowland areas with relatively low population density, followed by neighboring Bench Maji and Halaba. In addition to population density, the southern and western parts of SNNP are further from the political center of the region (in Sidama). Based on this regional level analysis, it is also probable that districts with less political power may have disproportionately had their land put up for lease (e.g. Dejene and Cochrane, 2021) (additional research, including from other regions, will also be needed to further assess if this applies to the sub-region as well).

**Figure 3:**
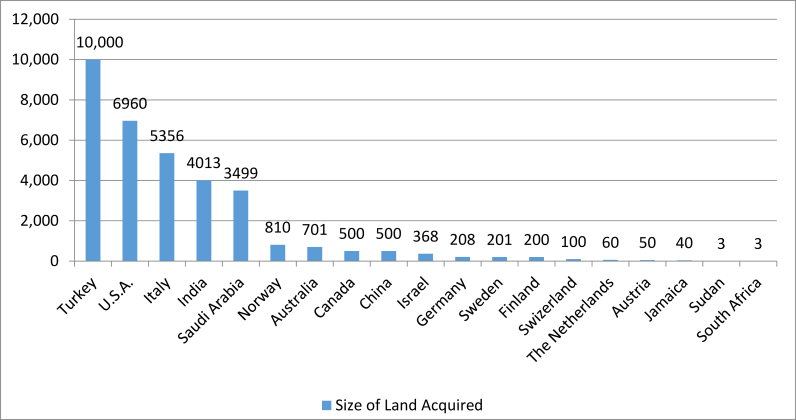
Countries of origin of foreign investors and size of holdings (Aggregated).

The 102 companies that have invested in SNNP are from diverse origin countries, the distribution of which differs with the findings of other studies conducted in Ethiopia. Many researchers (e.g. Lorenzo et al., 2011; Aisbett and Barbanente, 2016) claim that there is a shift from north-south to south-south investments, mainly because of the presence of many Indian investors in Ethiopia. In SNNP, however, in terms of the number of investors, the most number of foreign investors are American (as noted, Ethiopian investors hold the largest amount of land). However, while fewer in number of investors the largest amount of land is held by a Turkish investor, which acquired 10,000 ha in South Omo. This land is used to produce cotton and is at semi-operational level. The aggregate land holdings by country of origin are listed in Figure 3. SNNP appears to have its own microcosm of investment trends, which differ from the national trends. This reiterates that broad generalizations, even about nations, require nuance at sub-national levels; further research is needed to understand why variations of investor origin occur across regions.

**Figure 4:**
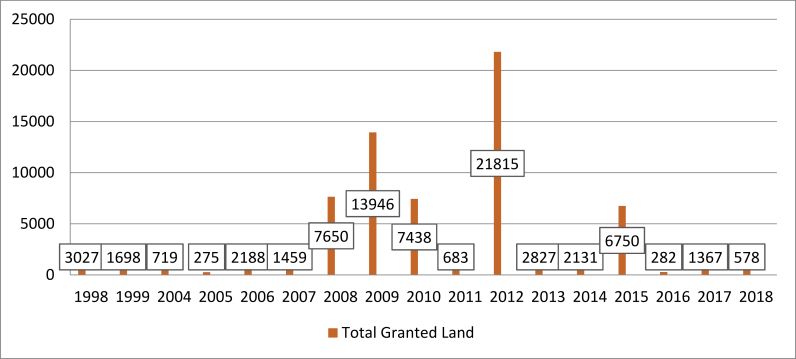
Size of land granted/leased over time.

In alignment with other research (e.g., Cochrane and Andrews, 2021), the land rush has occurred at different times in different places. This is also the case with Ethiopia, and it is also the case for sub-regions within the country. The specific peak years of investment for the country (as in Dejene and Cochrane, 2021) do not align with the peak years for SNNP (Figure 4). However, the broader narrative is the same: investment began in the late 1990s, rose sharply in the late 2000s and then declines after the regulatory reform of 2012 (Moreda, 2018). Variation in peak years is not necessarily reflective of different trends, as negotiation processes may be delayed or streamlined, as a result we do not delve into this divergence in detail.

**Figure 5:**
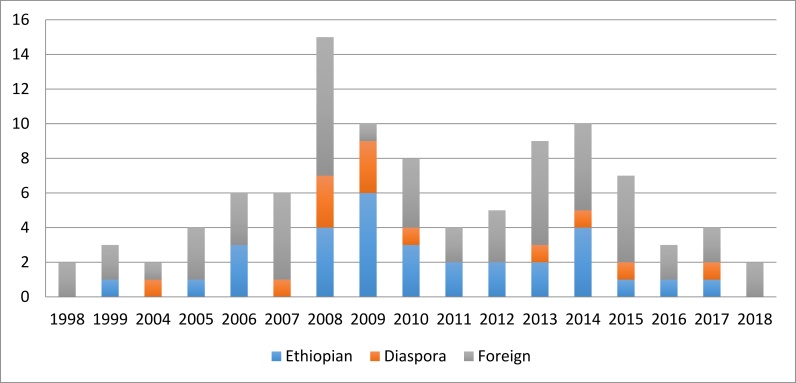
Type of investor by year of contract (by number of contracts).

Figure 5 shows the type of investor (Ethiopian, diaspora, foreign) over time, where we see the number of investors does not necessarily align with the amount of land granted. In other words, the rush for land by investors also occurred in waves, not just by aggregate land leased. Relative to their small number compared to the other two investor groups, diaspora investors are notably absent in most years, except in the years that followed the government calling for the Ethiopian diaspora to invest in their country (e.g., during the Ethiopian Millennium that took place in 2007, the years that followed). With regard to foreign investors, though it has been reported that their presence is minimal after the 2012 policy changes, we observe that foreign investors continued investing in the region after the regulatory changes. That contradicts what is commonly believed about the flow of FDI to the agricultural sector in Ethiopia (Rahmato, 2014; Dejene and Cochrane, 2021), arguments that have largely focused on trends of large-scale investments (5,000 ha and above). This shows that the land rush continued, although primarily with land leases below 1,000 ha, an investment trend that has received relatively limited attention in Ethiopia.

**Figure 6:**
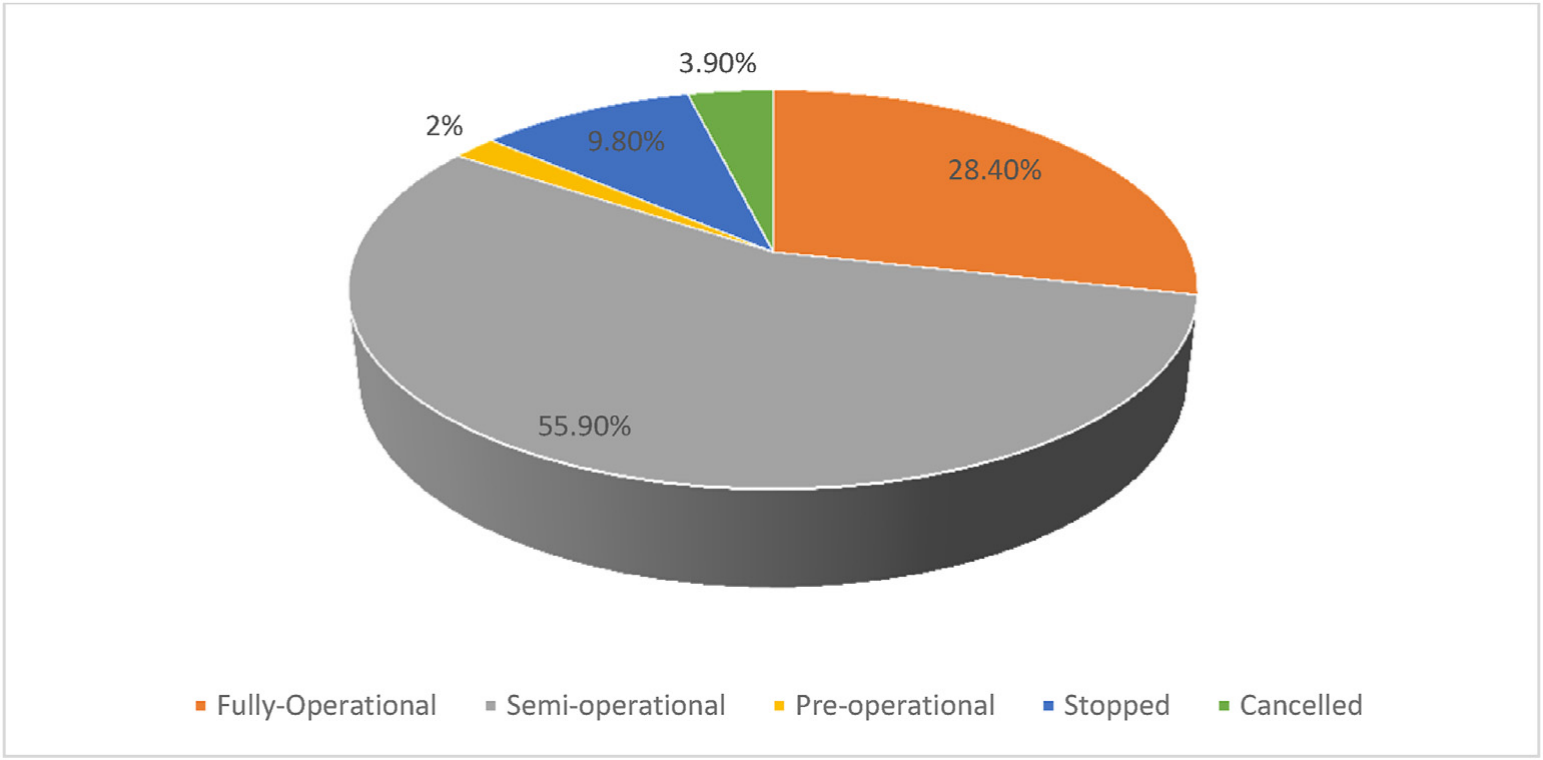
Percentage of investments by level of implementation.

Not all the granted/leased land has been utilized by investors; more than half is semi-operational and only 28.4% are fully operational (See Figure 6). As noted above, the Government of Ethiopia has struggled to ensure the leased land is used as it was granted for (as outlined in proposals and contracts), and is taking action against investors that are inactive or are only partially fulfilling their agreements. As reported by Alemu (2012) as well as Dereje et al. (2016), low levels of project implementation was a primary reason why the Ethiopian government ceased the transfer of land to investors in 2012 and was a driver for the series of policy revisions and cancellations that took place thereafter. Low levels of project implementation contribute to the lack of progress regarding expected positive spillovers that were promised both by the government and investors, an example of which is employment opportunities. However, from the perspective of investors, projects take time to implement, often requiring land clearing and infrastructural development, which may be done in phases. From the perspective of communities, the underutilized and unused lands are a cause of frustration and contestation (including instances of conflict), which contributes to slower project implementation due to an increased risk of failure.

Schoneveld (2015) claims that many of the investments that have taken place since 2006 are in the semi or pre-operational phases, a phase wherein investors have secured land but have not yet moved into the implementation or operation phases. According to the World Bank (2010), only 20% of investments in Sub-Saharan Africa have progressed to the farming stage. However, in a more recent study on large-scale commercial farm operations in Ethiopia, Wondimu (2016) identifies that 55% of land was being used. Our study aligns with the latter findings, showing that a majority of investments are semi-operational (which is not necessarily a signal of failure, it may mean that the investor is taking a phased approach to infrastructural development, which takes time to develop). Figure 6 shows that the majority (55.9%) of the investments in the region are in a semi-operational stage whereas only 28.4% are in the fully operational stage. Those that are in the pre-operational stage are investors that have not started any activity on the land, these only account for 2.0% and are largely newly contracted investments (both granted in 2018). The remainder of the investments have either stopped or been cancelled.

**Figure 7:**
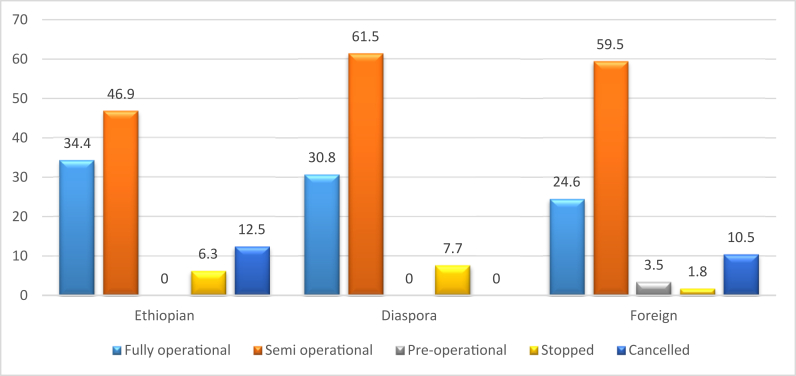
Percentage of investor at different implementation levels.

**Figure 8:**
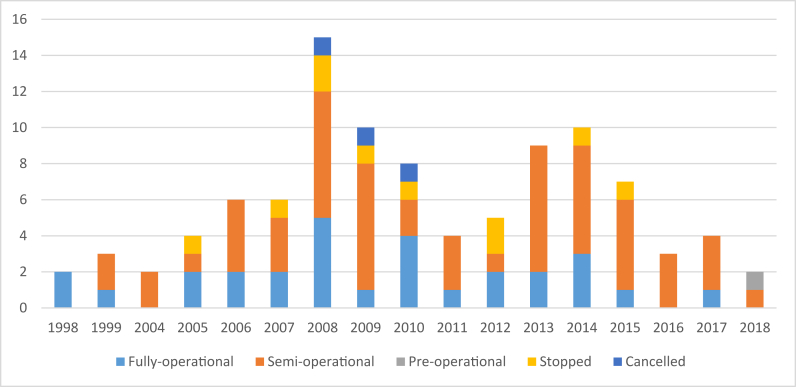
Level of implementation by year of contract (By number of deals).

Analyzing the investment status by investor type, we see some unique outcomes in comparing Ethiopian, foreign and diaspora investors (see Figure 7). Similar to the data shown in Figure 1, the rate of fully operational projects was highest among Ethiopian investors (34.4%), followed by diaspora investors (30.8%), and then foreign investors (24.6%). Interestingly, however, when the stopped and cancelled investments are combined, which we consider as unsuccessful or failed (pre-operational are excluded as these tend to be newer contracts, see Figure 8), we find that diaspora investors have the lowest rate of failure (7.7%) while Ethiopian and foreign investors have higher failure rates (18.8% and 12.3% respectively). For those that are semi-operational, diaspora investors have the highest rate (61.5%), followed by foreign investors (59.6%), and then Ethiopian investors (46.9%). This finding complicates generalizations about investors (including the findings shown in Figure 1), as while Ethiopian investors had larger holdings and greater shares of those holdings developed, they also have the highest rate of project failure (as shown in Figure 7). The Government of Ethiopia has been keen to promote domestic and diaspora investment, due to an apparent failure of foreign investment. This concern may be valid for the large investments granted by the federal government, however for the smaller investments at the regional level, foreign investors do not have a higher rate of failure and their fully- and semi-operational status is similar to that of Ethiopian and diaspora investors.

It is possible that implementation status is reflective of the date of contract, with contracts established at an earlier date having more time to become fully operational and/or more time for project failure to materialize. However, the cancelled and stopped deals are not the earliest; these deals took place between 2005 and 2015, with the cancelled deals being agreed between 2008 and 2012, suggesting that many investors rushed into agreements without sufficient information following the global commodity price spike, anticipating continued high prices. For example, one investment we have visited highlighted that the investor did not know the quality of the land that was being leased, which was water-logged for part of the year, making the intended crops not viable. The two investments that are at the pre-operational stage are those that were signed in 2018, which is the reason why they have not started yet. The absence of investments at the pre-operational stage in the earlier years is also an indicator that agricultural projects take time to establish and become operational as well as a signal that the Ethiopian government is seriously taking action with investors that are not implementing their projects according to their plans and contracts (be that real or perceived, with the high profile legal case surrounding the Karuturi investment in Gambella).

## 5 Job creation: by level of implementation, investor type and crop type

**Figure 9:**
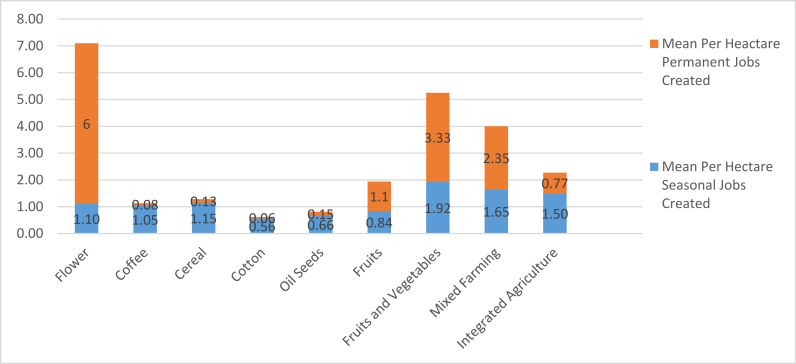
Mean per hectare job creation by crop type.

Much less addressed in the literature are factors that determine the amount of job creation as a result of agricultural investment. We assume that there would be a relationship between the types of crops and the number of jobs created, which is our first point of analysis using the data set of 102 investments. We then analyze investor type and implementation status in relation to crop type, also with a focus on employment creation. The number and type of jobs created are connected to the crop type grown, as some crops are more labor intensive while others require only seasonal labor. To assess the extent to which crop type impacts the amount and type of job creation, we analyzed these variables in relation to the average per hectare permanent and seasonal job creation (Figure 9). As assumed, job creation is equal for all crop types. For some crops (e.g., coffee, cereals, oil seeds, cotton, and integrated agriculture) seasonal job creation is higher than the mean per hectare permanent jobs created. For other crops (flowers/horticulture, fruits and vegetables, and mixed farming permanent) jobs creation is higher than the seasonal job creation.

**Figure 10:**
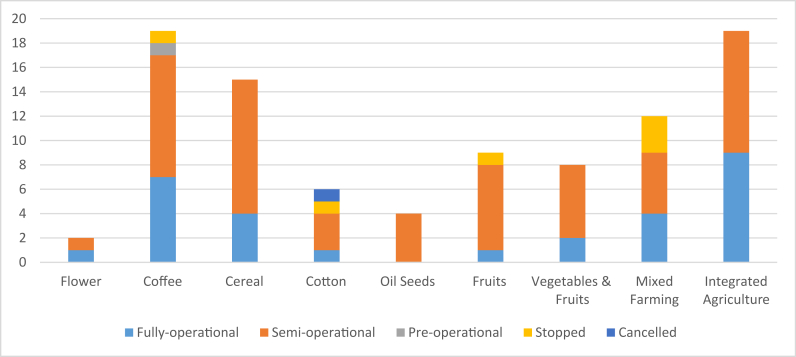
Implementation level by crop type.

**Figure 11:**
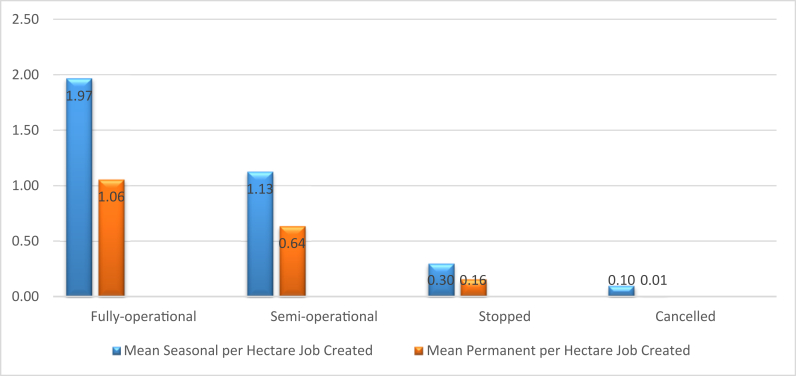
Mean per hectare jobs created by level of implementation.

An additional factor worthy of consideration in job creation is investment implementation status, and we find that most of the crops that are identified as creating the largest size of permanent jobs (flowers/horticulture, integrated agriculture and fruits and vegetables), are largely in the semi- or fully-operational levels (Figure 10). Figure 11 shows that as the level of implementation of investment goes down from fully operational to cancelled, the mean per hectare job created of both types decreases. In other words, while crop type is important for job creation, so too is implementation status.

**Figure 12:**
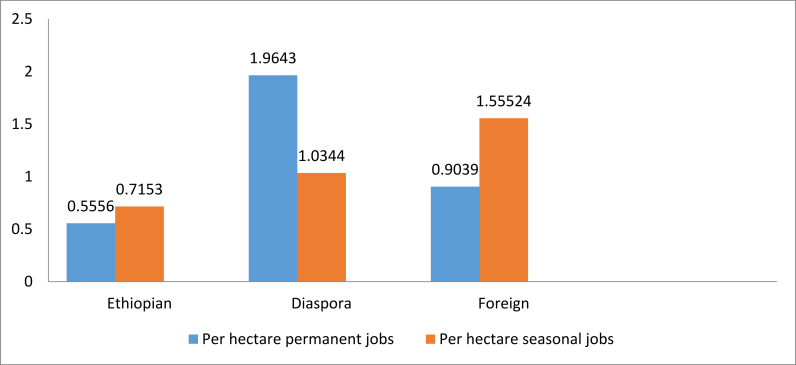
Per hectare job creation by investor type.

Furthermore, not all investments are equal in size. In order to assess the impact of land lease size on job creation we conducted analysis based on the per hectare job creation by investor types. This analysis included both seasonal and permanent jobs (Figure 12). Counter intuitively, given the above findings shown in Figures 1 and 7, Ethiopian investors have a lower per hectare employment creation of permanent and seasonal jobs than both the diaspora and foreign investors. This might be because Ethiopian investors are not integrating new approaches or technologies, and are instead leasing land and using traditional methods that are less intensive. Further analysis is required to assess if this indeed contributes to such an outcome; our initial site visits indicates that this might be one reason contributing to this finding.

### 5.1 Regression analysis of job creation, crop type and investor type

The potential for employment opportunities resulting from investment in agriculture has been utilized as a focal justification by the Government of Ethiopia and development banks to encourage and enable investors to acquire land, and thereby other resources such as water. But it is not yet known where, how, and in what condition, this objective could be realized. Available evidence suggests that job creation is minimal, and tends to be temporary (Alamirew et al., 2015). Based on the SNNP data set, we conducted a regression analysis to find where and how agricultural investments created jobs.

Previous research in Ethiopia has reported that the average rate of job creation by large-scale agricultural investments is 14 ha to one permanent job and 5.2 ha for one seasonal job (Degife and Wolfram, 2017). Using this rate we examined the number of jobs created in SNNP. The total leased land was 75,259 ha and the total number of permanent and seasonal jobs created is 6,118 and 23,552 respectively. According to our 2020 data, to create 1 permanent job, therefore, the government needed to transfer 12.3 ha of land to investors, and 3.2 ha of land to create a seasonal job. The rate identified using this SNNP data is better in comparison to the national average, and may be used as evidence to argue that investors in the region are doing better in creating employment opportunities or are engaging with investment types (e.g. crops) that are more labor intensive and create more jobs. The analysis implies that the job creation of the regional state is 113.8% for the permanent jobs and 162.7% for the seasonal jobs, compared to what was expected to be created, based on the national averages. The figure in this study is also far better than the finding of a study that was conducted in Oromia Regional State, where one permanent job was created from every 20 ha of land granted to an investor (Alamirew et al., 2015). The descriptive statistics of the data set shows that for the permanent jobs created by the 102 investments included in this study the mean/average number of permanent job created by the investments is 59.98 with its median being 9.00. The mode (the value with the highest frequency) is 0, as 12 of the 102 investments have created no permanent job at all. The smallest number of permanent job created is 0 and the highest permanent job created is 2001. Of all the 102 investments, 6,118 permanent jobs were created. As it is mentioned above in the methodology, the reason why we employed negative binomial regression instead of a Poisson regression model is because the data is dispersed. The variance of the data is 47,662.83 whereas the mean is only 59.98, a huge gap indeed. Based on these figures, we analyzed which investor types created more jobs and which crop types enabled greater employment opportunities (Table 3).

**Table 3:** Regression Analysis to compare among Investor Type and Crop Type for Job Creation.

Tests of Model Effects			
Source	Type III		
	Wald Chi-Square	df	Sig.
(Intercept)	26.556	1	.000
Investor Type	3.808	2	.149
Crop Type	121.765	9	.000

Parameter Estimates										
Parameter	B	Std. Error	95% Wald Confidence Interval		Hypothesis Test			Exp(B)	95% Wald Confidence Interval for Exp(B)	
			Lower	Upper	Wald Chi-Square	df	Sig.		Lower	Upper
(Intercept)	1.212	.7840	−.324	2.749	2.391	1	.122	3.362	.723	15.629
Foreign	−.048	.2628	−.563	.467	.034	1	.855	.953	.569	1.595
Diaspora	.694	.3998	−.090	1.477	3.010	1	.083	2.001	.914	4.381
Ethiopia	0^a^	.	.	.	.	.	.	1	.	.
Unknown	−2.650	.8599	−4.336	−.965	9.500	1	.002	.071	.013	.381
Inte. Agric.	−1.842	.7664	−3.344	−.340	5.775	1	.016	.159	.035	.712
Mixed Farming	−.979	.8404	−2.626	.668	1.356	1	.244	.376	.072	1.951
Veg. & Fruits	−.132	.8371	−1.773	1.508	.025	1	.874	.876	.170	4.519
Fruits	−2.166	.8139	−3.761	−.571	7.080	1	.008	.115	.023	.565
Oil Seeds	−3.050	.9151	−4.844	−1.256	11.107	1	.001	.047	.008	.285
Cotton	−4.041	.8672	−5.741	−2.341	21.714	1	.000	.018	.003	.096
Cereal	−3.267	.8088	−4.853	−1.682	16.319	1	.000	.038	.008	.186
Coffee	−3.767	.8008	−5.336	−2.197	22.123	1	.000	.023	.005	.111
Flower	0^a^	.	.	.	.	.	.	1	.	.
(Scale)	1^b^									
(Negative binomial)	1^b^									

Dependent variable: Number of permanent job created.Model: (Intercept), Investor type, Crop type, offset = Developed land.

Dependent variable: Number of permanent job created.Model: (Intercept), Investor type, Crop type, offset = lnplus.

a Set to zero because this parameter is redundant.

b Fixed at the displayed value.

As shown in Table 3, of the two predictors included in the model only crop type is statistically significant. The P-value of crop type is 0.00. However, when it comes to investor type (P-value of 0.149, well above 0.05), which makes the association between investor type and the per hectare job created non-significant. From this we conclude that there is no statistically significant difference among the three groups of investors in terms of job creation. The other comparison we conducted is between the ten types of crops. The crop that is used as a reference is flowers/horticulture (as we identified it creating the highest amount of per hectare jobs created). Since the Exponential Beta values of all the remaining crops are less than one, flower production created the largest number of jobs (in line with the data in Figure 9). Of the remaining nine crops vegetables and fruits created 87.6% of the permanent jobs created by the production of flower. Mixed farming, integrated agriculture, fruits, oil seeds, cereal, coffee and cotton follows with 37.6%, 15.9%, 11.5%, 4.7%, 3.8%, 2.3% and 1.8% respectively. This aligns with the findings shown in Figure 9. The results figures show that the production of flowers creates the largest number per hectare permanent jobs, followed by investments that produced vegetables and fruits.

Our null hypothesis (H_o_) (i.e. there is no difference among the investors and crop types in terms of job creation) is accepted for the investor type and rejected for the crop type. That means as the P-value of the first categorical predictor (investor type, is 0.149, well above 0.05) the alternative hypothesis (H_a_) (i.e. there is no difference among the three investor types in terms of permanent job creation) is accepted. When it comes to crop type, with a P-value 0.00 its null hypothesis (H_0_) is rejected, whereas, the alternative hypothesis (H_a_) (i.e. there is difference among the nine crop types in terms of permanent job creation) is accepted. That means there is a statistically significant difference among the nine types of crops harvested in terms of permanent job creation. Finally, because of the categorical nature of the predictors of the model, we can only compare among the categories of both variables. We cannot develop a mathematical model to be used for predicting the number of permanent jobs created by the production of different types of crops. What we do instead is compare among the nine crop types and discuss which ones created more and which ones less.

## 6 Conclusion

One of reasons that the Government of Ethiopia provided so many incentives to investors within the agricultural sector was to foster employment creation (alongside technology transfer, increasing productivity, and other aims). Much of the literature on agricultural investment has focused on case studies and comparative case studies, with limited evidence analyzing quantitative data to assess trends (and thereby avoid the biases of site selection with cases). This paper analyzes a dataset of 102 investments in one region of Ethiopia, and specifically looks at the differences amongst investments with regard to investor type, size of land lease, and crop type in order to differentiate the impacts on job creation.

The point of departure of this study is the assumption that not all investments are equal in terms of job creation. In testing whether this assumption is true or not we conduct both descriptive and inferential analysis on different attributes of the 102 investments. The results show some alignment with existing findings, and provide new perspectives that differ with what is commonly believed about agricultural investment in Ethiopia. For example, it has been commonly argued that domestic investors are larger in number but have acquired smaller areas of land (as individual contracts, and in many countries in the aggregate in comparison to foreign investors). For SNNP, we find that domestic investors are smaller in number than the foreign ones, and domestic investors have acquired larger average size of land (though the opposite could be true for larger investments that are granted by the federal government). In terms of the size of land developed, Ethiopian investors had developed the most, yet created the least mean per hectare job. Foreign investors were predominately originating from the northern hemisphere, contrary to the common assumption reflected in recent studies suggesting that there has been a shift of investments from north-south to south-south. While this may be the case in some regions of Ethiopia and other countries, this is not the case in this study region.

In line with other studies, semi-operational projects account for more than half of the investments followed by the fully operational ones. Regarding this, Ethiopian investors have the highest rate of both successful and failed projects, with diaspora investors having the lowest failure rate. The failure rate of foreign investors does not differ significantly from domestic investors, which contests part of the narrative presented by the Government of Ethiopia is restricting foreign investors after the 2012 policy reform. Rather than investor type, we find the temporal factor more important for failure, with earlier investments (those occurring before 2007/8) being more successful, while those leasing land during the ‘rush’ following the 2007/8 period (up to 2012) experienced higher levels of failure.

Of the attributes that are included in the job creation analysis (implementation status, crop type, and investor type), we find variation in the mean per hectare job created based on crop type. Crops like fresh cut flowers, vegetables and fruits, and mixed farming created the highest mean per hectare permanent jobs, whereas coffee, cereals, and oil seeds created higher mean per hectare seasonal jobs. On the same topic of crop type, our regression analysis revealed that there is a statistically significant difference among the ten categories of crops included in this study. Horticulture created the highest per hectare permanent jobs, followed by vegetables and fruits. Our investigation of investor type as a determining factor reveals that even though the descriptive analysis shows differences among the three types of investors in their capacity to create employment opportunity, the result is found to be not statistically significant.

## Declarations

### Author contribution statement

Habiba Mohammed Yimam: Conceived and designed the experiments; Performed the experiments; Analyzed and interpreted the data; Wrote the paper.

Logan Cochrane: Conceived and designed the experiments; Analyzed and interpreted the data; Wrote the paper.

Melisew Dejene Lemma: Conceived and designed the experiments; Analyzed and interpreted the data.

### Funding statement

This research did not receive any specific grant from funding agencies in the public, commercial, or not-for-profit sectors.

### Data availability statement

Data will be made available on request.

### Declaration of interests statement

The authors declare no conflict of interest.

### Additional information

No additional information is available for this paper.
